# Unconventional Secretion of Tissue Transglutaminase Involves Phospholipid-Dependent Delivery into Recycling Endosomes

**DOI:** 10.1371/journal.pone.0019414

**Published:** 2011-04-27

**Authors:** Evgeny A. Zemskov, Irina Mikhailenko, Ru-Ching Hsia, Liubov Zaritskaya, Alexey M. Belkin

**Affiliations:** 1 Department of Biochemistry and Molecular Biology, University of Maryland School of Medicine, Baltimore, Maryland, United States of America; 2 Center for Vascular and Inflammatory Diseases, University of Maryland School of Medicine, Baltimore, Maryland, United States of America; 3 Department of Physiology, University of Maryland School of Medicine, Baltimore, Maryland, United States of America; 4 Marlene and Stewart Greenebaum Cancer Center, University of Maryland School of Medicine, Baltimore, Maryland, United States of America; 5 Center for Stem Cell Biology and Regenerative Medicine, University of Maryland School of Medicine, Baltimore, Maryland, United States of America; 6 Core Imaging Facility, University of Maryland Dental School, Baltimore, Maryland, United States of America; 7 Applied and Developmental Research Support Program, Science Applications International Corporation, Frederick, Maryland, United States of America; University of Nebraska Medical Center, United States of America

## Abstract

Although endosomal compartments have been suggested to play a role in unconventional protein secretion, there is scarce experimental evidence for such involvement. Here we report that recycling endosomes are essential for externalization of cytoplasmic secretory protein tissue transglutaminase (tTG). The *de novo* synthesized cytoplasmic tTG does not follow the classical ER/Golgi-dependent secretion pathway, but is targeted to perinuclear recycling endosomes, and is delivered inside these vesicles prior to externalization. On its route to the cell surface tTG interacts with internalized β1 integrins inside the recycling endosomes and is secreted as a complex with recycled β1 integrins. Inactivation of recycling endosomes, blocking endosome fusion with the plasma membrane, or downregulation of Rab11 GTPase that controls outbound trafficking of perinuclear recycling endosomes, all abrogate tTG secretion. The initial recruitment of cytoplasmic tTG to recycling endosomes and subsequent externalization depend on its binding to phosphoinositides on endosomal membranes. These findings begin to unravel the unconventional mechanism of tTG secretion which utilizes the long loop of endosomal recycling pathway and indicate involvement of endosomal trafficking in non-classical protein secretion.

## Introduction

A great majority of proteins localized on the cell surface and in the ECM are transported outside through the classical ER-Golgi pathway for which the key mechanisms of molecular recognition and trafficking have been established [1], [2]. Yet, there are several proteins that are found in the extracellular space, but do not have leader sequence or hydrophobic domains, do not localize to the ER/Golgi, and lack posttranslational modifications generated in these compartments [3]-[8]. Among them, some have primary function(s) outside the cell, while others function both intra- and extracellularly [6], [7].

Several mechanisms were proposed to function in non-classical protein secretion. The first, exemplified by externalization of fibroblast growth factor 2 (FGF2), is characterized by phospholipid-mediated targeting and direct translocation of this protein across the plasma membrane [9]. The second is based on sequestration of cytoplasmic proteins such as interleukin-1β (IL-1β) by secretory lysosomes and their subsequent inflammation-mediated release into the extracellular space [10], [11]. Two other pathways involve microvesicle-dependent secretion and include either shedding of vesicles at the plasma membrane or formation of endosomal intraluminal vesicles/multivesicular bodies that release internal vesicles outside the cell upon their fusion with the plasma membrane [12], [13]. Also, a recently described non-classical pathway was reported to depend on autophagosomes [14], [15]. Surprisingly, caspase 1 and GRASP were found to control several unconventional secretion routes, indicating some shared steps in the diverse pathways of non-classical secretion [16], [17]. Despite this progress, general mechanisms and specific molecular requirements for trafficking pathways of unconventional secretion remain to be elucidated.

tTG is a ubiquitous member of the transglutaminase family of Ca^2+^-dependent cross-linking enzymes which also possesses GTPase, disulfide isomerase and protein kinase activities [18], [19]. While the majority of tTG pool is present in the cytoplasm, and some amounts are found in the mitochondria and nucleus, no tTG is detected in the ER or Golgi [18]. Depending on cell type, a significant tTG fraction (1–20%) is localized on the plasma membrane and in the ECM [19]. tTG has both enzymatic and non-enzymatic functions at these locations where it cross-links ECM proteins and modulates the interactions of cells with the ECM and growth factors by non-covalent regulation of integrins [20]–[22], syndecan-4 [23]–[25], and growth factor receptors [26]. Mounting data suggest that tTG has common or related functions inside and outside the cells, such as regulation of cell survival [7]–[19].

tTG is constitutively externalized from undamaged cells and fibroblasts, osteoblasts, endothelial, smooth muscle cells, and monocytes/macrophages, all contain this protein on their surface and in the ECM [18]. There is no secretory signal or hydrophobic/transmembrane domains in tTG [27]–[28] and nothing is known regarding the factors that control its secretion. While many agents regulate cellular tTG levels, biosynthesis, and degradation, they all concurrently modulate its levels outside the cell [18], [19], suggesting a default pathway for trafficking this protein to the cell surface. A significant part of the tTG pool is present in the so-called “particulate fraction” indicating its association with membranes [18]. The causes of such association are unclear. It may depend on stable interactions of tTG with adrenergic receptors [29] or integrins [20]. Otherwise, a direct or indirect binding to lipids may target this protein to cell membranes. Two early studies reported *in vitro* association of tTG with phospholipids [30], [31], however no molecular basis or *in vivo* evidence of such interaction was presented. Although fibronectin and heparan sulphate proteoglycans, two extracellular binding partners of tTG, and its own transamidating activity, were all proposed to affect its secretion [25], [32], [33], they likely impact the retention of tTG on the surface rather than its outbound trafficking inside the cell.

In this study, we focus on the intracellular trafficking of tTG on its route to the cell surface. We demonstrate that the ER/Golgi-independent trafficking pathway of tTG externalization involves phosphoinositide-dependent recruitment of cytoplasmic protein to the perinuclear recycling compartment (PNRC), its delivery inside these vesicles, their outbound trafficking, and their fusion with the plasma membrane which releases tTG onto the cell surface. These results reveal an unexpected role of recycling endosomes in the unconventional secretion of cytoplasmic tTG.

## Results

### Kinetics of tTG Externalization and Deposition in the ECM

**Figure 1:**
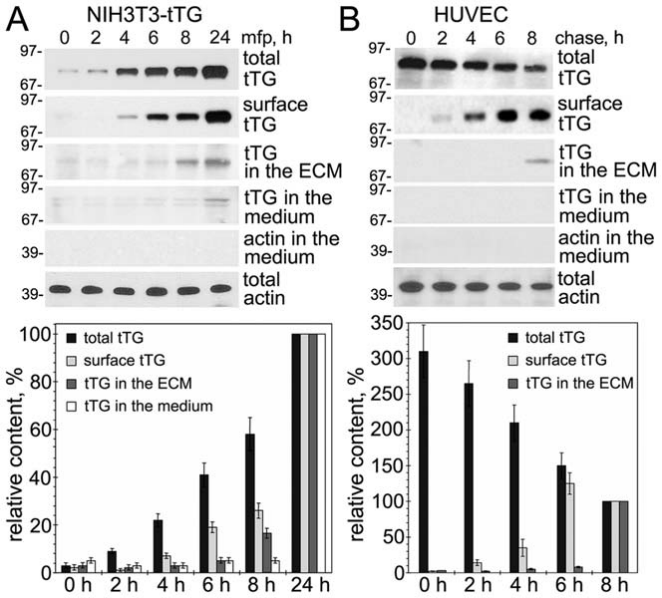
Kinetics of tTG Secretion and Deposition into the ECM. (A) Externalization of exogenous tTG in NIH3T3-tTG fibroblasts. The cells were induced to synthesize tTG with mifepristone. tTG on the cell surface was detected by labeling cells with sulpho-NHS-LC-biotin, isolation of cell surface proteins on Neutravidin-Agarose, and immunoblotting. tTG was also immunoprecipitated from the ECM and growth medium and analyzed by immunoblotting. Total tTG and β-actin levels in NIH3T3-tTG fibroblasts were determined by direct immunoblotting. The relative tTG amounts in each cell fraction were quantified and compared to those at 24 h of induction. (B) Externalization of endogenous tTG in HUVECs. Cells were labeled with ^35^S-Translabel and then chased for indicated time with medium containing no radioactivity. The *de novo* synthesized tTG in cell fractions was analyzed by its immunoprecipitation from whole cell extracts, cell surface protein fraction, the ECM and growth medium, followed by SDS-PAGE and fluorography. The relative tTG amounts in cell fractions were quantified by scintillation counting and compared to those at 8 h of chase. The lack of β-actin in culture medium was tested by blotting (A) or by immunoprecipitation, SDS-PAGE and fluorography (B), and showed the absence of cell lysis. Shown in (A,B) are representative of three independent experiments. Bars in (A,B) depict means ± SEM. See also Figure S1.

To study the kinetics of tTG secretion, we employed NIH3T3-tTG transfectants in which expression of exogenous tTG is silent unless is induced by mifepristone (Fig. 1A, [26], [34]). After induction of tTG synthesis, its content was evaluated in the whole cell extracts, on the cell surface after isolation of biotinylated (surface) tTG, in the ECM, and in the growth medium. tTG was detected on the cell surface ∼4 h after onset of biosynthesis and its amounts continued to increase thereafter. In contrast, tTG deposited in the ECM became detectable ∼8 h, and in the medium - ∼24 h after induction. A similar kinetics of tTG externalization and deposition in the ECM was observed in HUVECs that express endogenous protein (Fig. 1B). In HUVECs, the *de novo* synthesized tTG was found on the surface ∼2 h, and in the ECM - ∼8 h after onset of its synthesis, but was not yet detected in the medium. Hence, tTG is not secreted as soluble protein and then binds back to the cell surface and the ECM as proposed earlier [32], but appears first on the outside leaflet of the plasma membrane and only later is translocated to the ECM. Blocking dynamin-dependent endocytosis of tTG from the surface [34] excluded a possibility that internalization hinders the ability of its detection at early time points of biosynthesis and revealed a dynamic equilibrium between the secretion of tTG and its removal from the cell surface (Fig. S1).

### tTG Secretion Follows the ER/Golgi-Independent Pathway which Requires Intracellular Membrane Fusion and Is Stimulated by Ca^2+^

**Figure 2:**
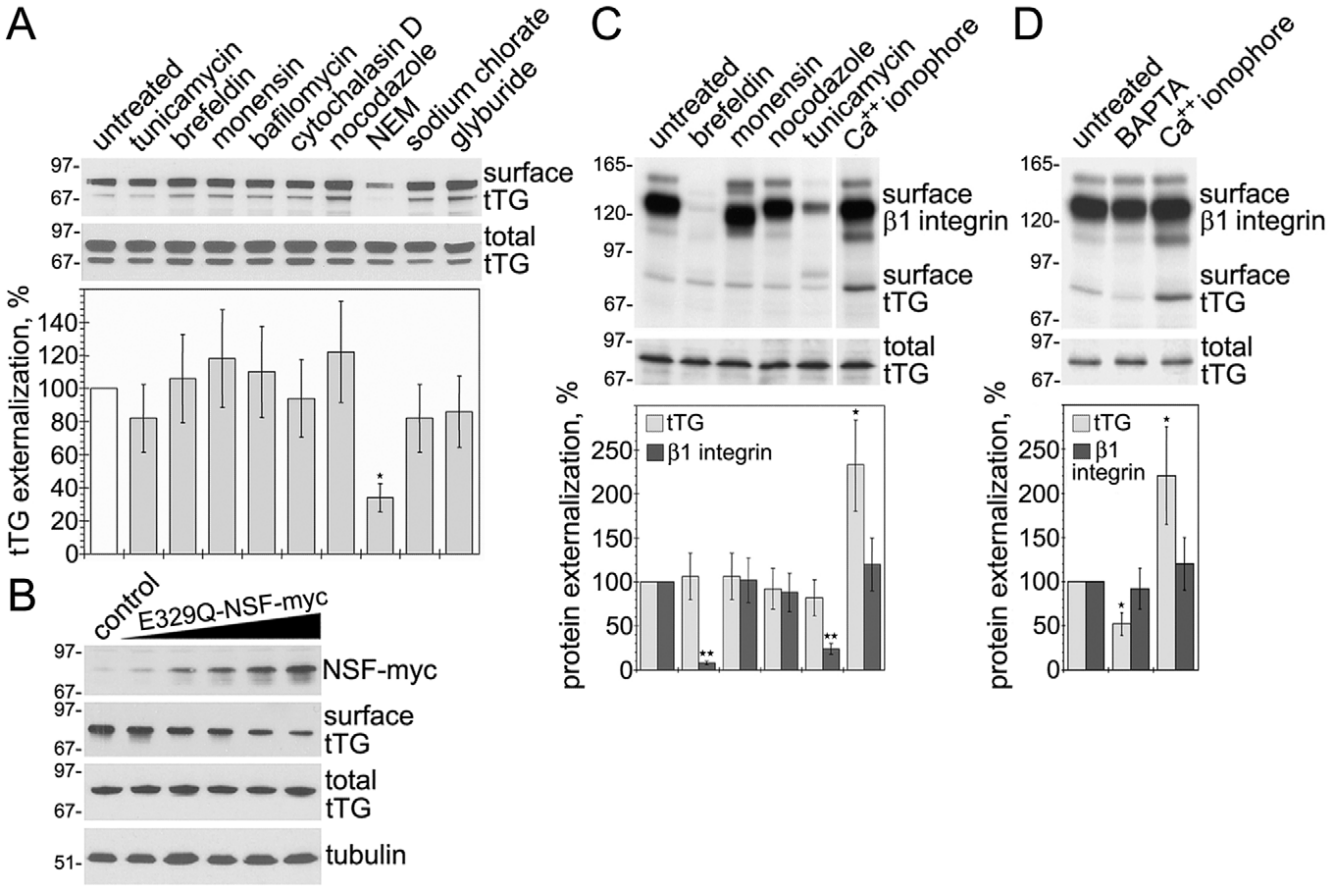
Externalization of tTG Does Not Involve the Classical ER/Golgi Secretion Pathway, but Requires Membrane Fusion and is Stimulated by Ca^2+^. (A) Non-classical secretion of tTG in NIH3T3-tTG fibroblasts. Cells were treated with inhibitors and then induced for 4 h with mifepristone before cell surface biotinylation and isolation of surface proteins. Cell surface and total tTG levels were defined by immunoblotting. The relative surface tTG levels were quantified and compared to that in untreated cells. (B) NSF-mediated membrane fusion is required for tTG secretion in NIH3T3-tTG fibroblasts. Cells were transiently transfected with increasing amounts of plasmid encoding the myc-tagged E329Q dominant negative NSF mutant. After 48 h, the transfectants were induced to synthesize tTG for 4 h. The levels of E329Q-NSF-myc were defined by immunoblotting with anti-myc antibody. The total and surface levels of tTG were determined as in (A). (C) Non-classical secretion of tTG in HUVECs. Cells were treated with inhibitors and then labeled for 6 h with ^35^S-Translabel before cell surface biotinylation and isolation of surface proteins. (D) Intracellular Ca^2+^ levels regulate tTG externalization in WI-38 fibroblasts. Cells were treated for 1 h with BAPTA or Ca^2+^ ionophore and then labeled for 6 h with ^35^S-Translabel before cell surface biotinylation and isolation of surface proteins. Cell surface levels of *de novo* synthesized tTG and β1 integrin and total levels of tTG in (C,D) were defined by immunoprecipitation, SDS-PAGE and fluorography. The relative surface levels of tTG and β1 integrin were quantified by scintillation counting and compared to those in untreated cells. Shown in (A–D) are representative of three independent experiments. Bars in (A,C,D) depict means ± SEM, *p<0.05, **p<0.005. See also Figure S2.

To establish whether trafficking of this protein to the surface of NIH3T3-tTG fibroblasts follows the classical ER/Golgi route, we employed pharmacologic inhibitors that affect the state of various cell organelles and cytoskeleton (Fig. 2A). Most of these inhibitors, including brefeldin and tunicamycin which block the ER/Golgi-dependent secretion, failed to interfere with the transport of tTG to the surface. Notably, sodium chlorate, a biosynthetic inhibitor of heparan sulfate proteoglycans, which blocks the non-classical secretion of FGF2 [35], and glyburide, an inhibitor of non-classical IL-1β secretion [10], did not affect tTG externalization. Further, neither heat shock nor Cu^2+^ chelator tetrathiomolybdate that regulate ER/Golgi-independent secretion of FGF1 [36], altered the externalization of tTG (Fig. S2). The only exception was *N*-ethylmaleimide (NEM), a specific inhibitor of NSF ATPase that is required for most intracellular membrane fusion events [37], which reduced the rate of tTG externalization. Also, transient expression of dominant negative E329Q-NSF mutant [38] in NIH3T3-tTG cells inhibited tTG secretion (Fig. 2B). The requirement for NSF-mediated membrane fusion and kinetics of tTG externalization (Fig. 1) indicate that the protein maintains its association with membranes during trafficking to the cell surface. Similarly, in HUVECs, neither brefeldin nor tunicamycin, which interfered with secretion of β1 integrin via the classical ER/Golgi route, affected the tTG secretion (Fig. 2C). Thus, tTG and its surface binding partner β1 integrin are externalized via independent secretory routes. The stimulatory effect of Ca^2+^ on tTG secretion observed in HUVECs was confirmed with WI-38 fibroblasts that also synthesize endogenous protein (Fig. 2D). Whereas BAPTA decreased the rate of tTG externalization, Ca^2+^ ionophore promoted the secretion of tTG. Thus, delivery of intracellular tTG to the cell surface does not require the ER/Golgi function and occurs via a non-classical secretion route which involves intracellular membrane fusion and is promoted by Ca^2+^.

### Cytoplasmic tTG Is Recruited to Perinuclear Recycling Endosomes and Delivered Inside these Vesicles Prior to Externalization

**Figure 3:**
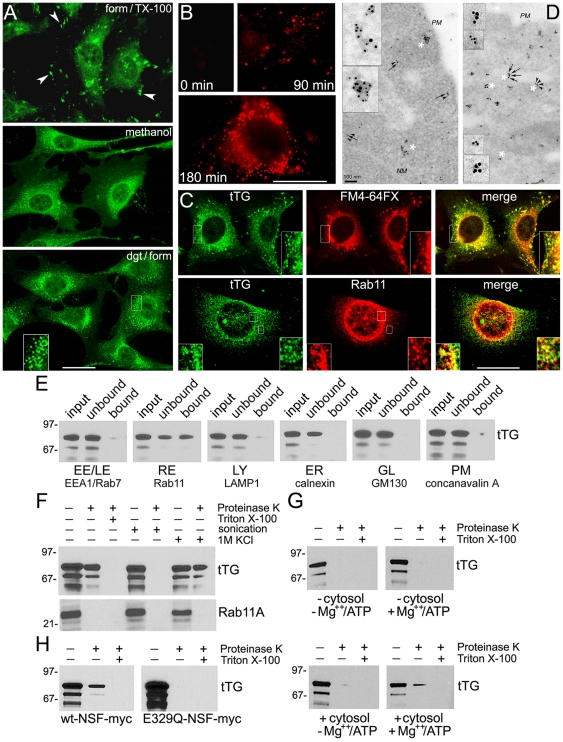
Intracellular tTG Is Associated with Perinuclear Recycling Endosomes and Delivered Inside these Vesicles Prior to Externalization. (A) WI-38 fibroblasts were fixed and permeabilized with formaldehyde and Triton X-100, fixed/permeabilized with ice-cold methanol, or permeabilized at 4°C with digitonin in QP buffer before fixation (see Materials and Methods). Note the localization of tTG in focal adhesions (*arrowheads*) and its accumulation in perinuclear vesicles (*insert*). (B) NIH3T3-tTG fibroblasts induced to synthesize tTG for 0–3 h, were extracted with digitonin before fixation and then stained for tTG. Immunofluorescence in (A,B) was analyzed by conventional microscopy. Scale bars - 10 µm. (C) NIH3T3-tTG fibroblasts were induced to synthesize tTG for 3 h, then labeled with lipophylic dye FM4-64FX, washed, extracted with digitonin, fixed, and stained for tTG. The digitonin-extracted cells were also double-stained for tTG and the marker of recycling endosomes, Rab11. Note a significant co-localization of tTG with perinuclear membranes and Rab11 (*inserts*). Immunofluorescence was analyzed by laser confocal microscopy. Scale bar - 10 µm. (D) Immunoelectron microscopic localization of tTG in NIH3T3-tTG fibroblasts 3 h after induction of synthesis. Double labeling of thin sections was performed for tTG (6 nm gold, *arrowheads*) and Rab11 (10 nm gold, *arrows*, left panel) or lysosomal marker Lamp3 (10 nm gold, *arrows*, right panel). Asterisks mark selected vesicles shown as inserts at higher magnifications. *NM* - nuclear membrane; *PM* - plasma membrane. Note a co-distribution of tTG and Rab11 on the vesicles and a general lack of thereof for tTG and Lamp3. (E) Intracellular tTG is associated with recycling endosomes. NIH3T3-tTG fibroblasts were induced to synthesize tTG for 3 h. Early/late endosomes (EE/LE), recycling endosomes (RE), lysosomes (LY), ER, Golgi (GL), and plasma membrane (PM) vesicles were isolated from crude membrane fraction using magnetic beads coated with antibodies to organelle markers. tTG levels were determined by SDS-PAGE and immunoblotting. Note the enrichment of tTG in recycling endosomes and its low levels - in early/late endosomes and plasma membrane. (F) tTG is present inside the recycling endosomes prior to externalization. The recycling endosomes isolated from NIH3T3-tTG fibroblasts 3 h after induction of tTG synthesis were treated at 4°C with proteinase K, with or without Triton X-100, sonication or high salt. (G) tTG delivery inside the endosomes requires ATP and cytosolic factor(s). Recycling endosomes immunoisolated from NIH3T3 fibroblasts lacking tTG were incubated for 1 h at 4°C with exogenous tTG, washed, warmed to 37°C for 1 h in the presence or absence of Mg^2+^/ATP and cytosol from the cells lacking tTG, and then treated with proteinase K with or without Triton X-100. (H) Membrane fusion is required for tTG delivery inside the recycling endosomes. NIH3T3-tTG fibroblasts were transfected with myc-tagged wild type NSF or its dominant negative E329Q mutant and induced to synthesize tTG for 3 h. Recycling endosomes were isolated from the transfectants and treated with proteinase K with or without Triton X-100. The contents of tTG (E–H) and Rab11A (F) in the vesicles were defined by immunoblotting. Shown in (E–H) are representative of three independent experiments. See also Figure S3 and Fig. S4.

We used immunostaining to examine a potential association of tTG with intracellular membrane compartments (Fig. 3A). In agreement with previous report [20], general staining for tTG allowed detection of this protein in focal adhesions and cytoplasm of WI-38 fibroblasts. In contrast, methanol fixation revealed accumulation of tTG around the nucleus. Pre-extraction of live cells with digitonin before fixation removed most cytoplasmic tTG and allowed to observe distinct tTG localization in perinuclear vesicles. Yet, we reported endolysosomal localization of internalized tTG [34]. Thus, to define intracellular localization of tTG prior to externalization, we used pre-extraction of majority of cytoplasmic tTG in NIH3T3-tTG fibroblasts shortly after onset of its synthesis and before the protein reached the surface (Fig. 3B). Whereas no tTG was detected before induction, the earliest staining was observed ∼1.5 h, and extensive localization at perinuclear vesicles - ∼3 h after initiation of tTG synthesis. Likewise, the localization of tTG in focal adhesions (Fig. S3A) and the perinuclear vesicles (Fig. S3B) was observed in NIH3T3-tTG-His/myc fibroblasts that were induced to express His/myc-tagged tTG [39], by immunostaining with antibody against the 6xHis tag.

Labeling of NIH3T3-tTG fibroblasts with styryl dye FM4-64FX 3 h after induction of tTG synthesis, followed by pre-extraction of cells and immunostaining for tTG with confocal microscopy, revealed a significant overlap between tTG localization and perinuclear membrane compartments (Fig. 3C). This indicated that *de novo* synthesized tTG is targeted to the PNRC prior to externalization. Double staining of these cells for tTG and the recycling endosomal marker Rab11, the late endosomal marker Rab7, and the lysosomal marker Lamp1, allowed to observe a substantial colocalization of tTG with Rab11 on a subset of perinuclear vesicles (Fig. 3C), whereas little codistribution of tTG with Rab7 or Lamp1 was found (Fig. S3C). Finally, using double immunogold labeling of thin sections of the NIH3T3-tTG fibroblasts early after induction of tTG synthesis, and electron microscopy, we found a prominent colocalization of tTG with Rab11 in the recycling endosomes (Fig. 3D) and presence of tTG on intralumenal vesicles of multivesicular endosomes (Fig. S3D), but observed little co-distribution of tTG with the lysosomal marker Lamp3. The localization of tTG on the intralumenal endosomal vesicles suggested that ESCRT-dependent inward budding of the limiting endosomal membrane might be involved in tTG secretion. However, shRNA-mediated depletion of ESCRT proteins Tsg101 and Vps24 did not affect tTG externalization, indicating that ESCRT function is dispensable for intravesicular delivery of cytoplasmic tTG (Fig. S4).

To biochemically ascertain the content of tTG in various cell membranes, we isolated a crude fraction of light and medium membranes [10], [11] 3 h after tTG induction in NIH3T3-tTG fibroblasts and used it for immunoaffinity isolation of vesicular organelles [40]–[42]. Biochemical analysis of immunoisolated membranes showed an enrichment of tTG in Rab11-containing recycling endosomes and its low content in other membrane types early after induction of its biosynthesis (Fig. 3E). The sidedness of newly synthesized tTG in relation to the Rab11-containing vesicles was examined by proteinase protection assays (Fig. 3F, [10]). Without detergent, proteinase K eliminated ∼70% of tTG in recycling endosomes, whereas ∼30% of this tTG pool was protected from degradation. In control experiments, all Rab11A outside the vesicles was digested with proteinase K even in the absence of detergent, while both tTG and Rab11A were completely degraded upon addition of detergent. Further, proteinase K eliminated all the tTG and Rab11A in recycling endosomes after their sonication, whereas high salt treatment left a significant fraction of tTG undegraded, proving that this latter part of endosomal tTG pool is present inside the vesicles. Therefore, cytoplasmic tTG binds to and is delivered inside the Rab11-containing PNRC shortly after onset of its biosynthesis but prior to externalization.

We also tested a delivery of purified recombinant tTG inside the recycling endosomes isolated from NIH3T3 fibroblasts lacking tTG (Fig. 3G). After incubation of tTG with recycling endosomes at 4°C, the protein bound to the vesicles. Their treatment with proteinase K without detergent revealed a complete degradation of vesicle-bound tTG, showing a lack of its delivery inside the vesicles in the absence of additional factors. While separate addition of Mg^2+^/ATP or cytosol from NIH3T3 cells lacking tTG did not produce any protease-protected tTG, their combination led to emergence of some tTG resistant to proteolysis. Hence, the delivery of endosome-bound tTG inside these vesicles requires ATP and some cytosolic factor(s). Finally, the proteinase protection experiments were repeated with recycling endosomes isolated from NIH3T3-tTG fibroblasts that expressed either wild type NSF or its E329Q dominant negative mutant (Fig. 3H). Inactive NSF mutant increased the overall tTG content on the recycling endosomes but abolished tTG delivery inside these vesicles. Thus, NSF and vesicular fusion are required for the delivery of endomembrane-bound tTG inside the recycling endosomes.

### Endosome Inactivation, Blocking Endosome Fusion with the Plasma Membrane, and Interference with the Long Loop of Endosomal Recycling, all Abrogate tTG Secretion

**Figure 4:**
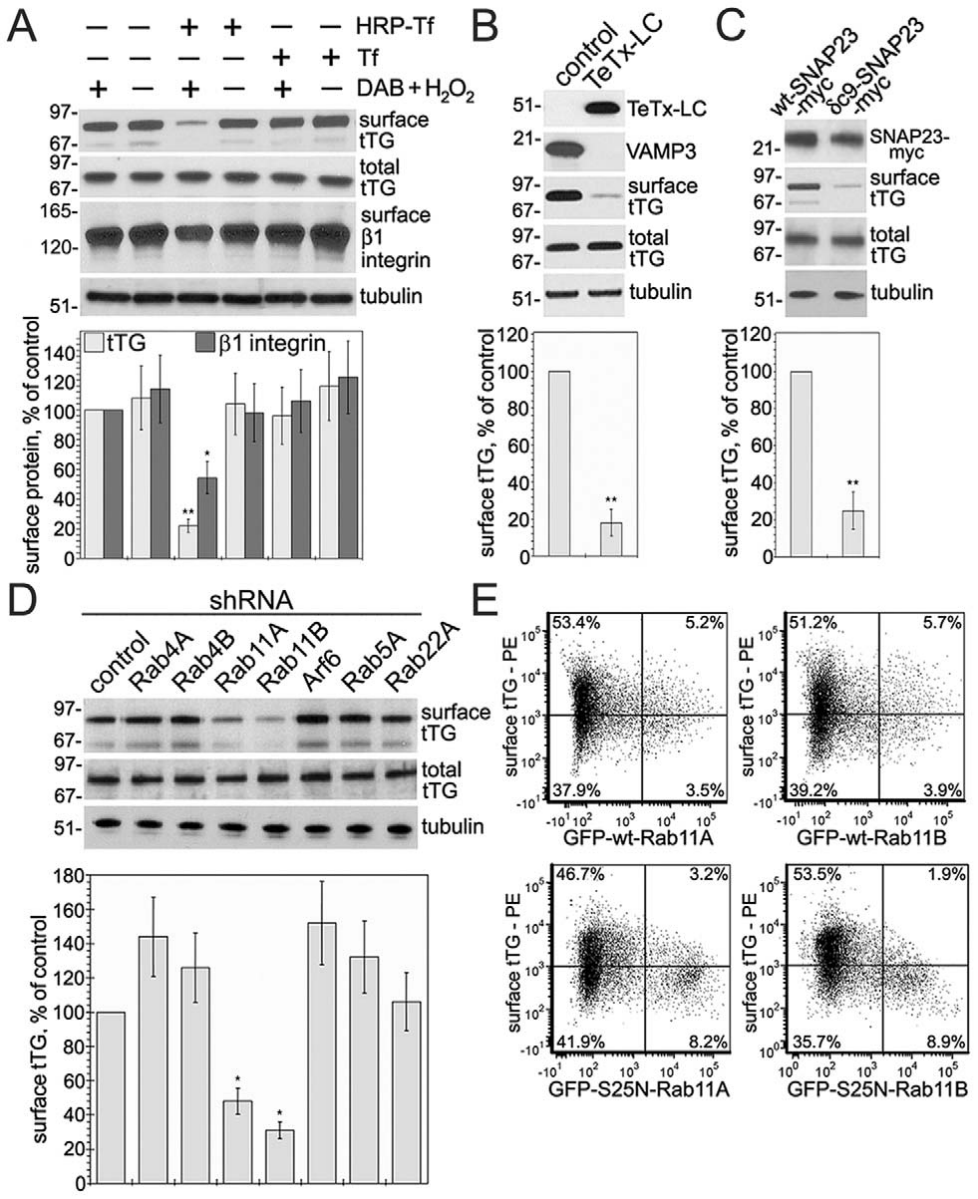
Endosome Ablation, Interference with Endosome to Plasma Membrane Fusion, and locking Rab11-Mediated Endosomal Recycling, all Inhibit tTG Externalization. (A) Functional inactivation of recycling endosomes inhibits tTG secretion. NIH3T3-tTG fibroblasts were incubated with HRP-transferrin or HRP, followed by DAB/H_2_O_2_ treatment, to ablate the recycling endosomes or lysosomes, respectively [43]. (B) Proteolysis of VAMP by *Tetanus* toxin inhibits tTG secretion. The light (catalytic) chain of *Tetanus* toxin (TeTx-LC) was expressed in NIH3T3-tTG fibroblasts. The TeTx-LC and VAMP3 levels were tested by immunoblotting. (C) Interference with SNAP23 function reduces tTG secretion. Wild type SNAP23 and its dominant negative δc9-SNAP23 mutant were expressed in NIH3T3-tTG fibroblasts as myc-tagged proteins and their levels were defined by immunoblotting. (D) Downregulation of Rab11A/Rab11B inhibits tTG secretion. shRNAs for Rab4A/B, Rab11A/B, Arf6, Rab5A and Rab22A and scrambled control were expressed in NIH3T3-tTG fibroblasts. tTG synthesis in (A–D) was induced for 4 h prior to cell surface biotinylation and isolation of surface proteins. Cell surface and total levels of tTG, β1 integrin and tubulin were defined by immunoblotting. The relative surface levels of tTG (A–D) and β1 integrin (A) were compared to those in DAB/H_2_O_2_-treated cells (A) or control transfectants (B–D). Shown in (A–D) are representative of three independent experiments. Bars show means ± SEM, *p<0.05, **p<0.005. (E) Interference with GTPase activity of Rab11 decreases tTG externalization. NIH3T3-tTG fibroblasts were transfected with wild type Rab11A, Rab11B, or their S25N dominant negative mutants fused to GFP. 48 h later the transfectants were induced to synthesize tTG for 4 h and live cells were stained at 4°C for surface tTG. Two-color flow cytometry of surface tTG (phycoerythrin) and transfected Rab11 proteins (GFP) levels is shown for gated live transfectants. Note a reduction in surface tTG levels in the transfectants that express high levels of Rab11A and Rab11B dominant negative mutants (right quadrants). Shown is representative of four independent experiments.

In order to assess the overall contribution of recycling endosomes to the process of tTG secretion, we utilized ablation of this compartment based on the formation of insoluble precipitate of horseradish peroxidase-transferrin (HRP-Tf) conjugates by cross-linking with diaminobenzidine (DAB) and hydrogen peroxide (Fig. 4A, [43]). Functional inactivation of recycling endosomes prior to tTG induction in NIH3T3-tTG fibroblasts reduced its surface level without altering the overall content of the protein, and also decreased the surface level of β1 integrin which is recycled through the PNRC [44], [45]. In contrast, inactivation of lysosomes by accumulation of free HRP and its cross-linking with DAB and hydrogen peroxide had no effect on tTG secretion or β1 integrin recycling. Therefore, recycling endosomes represent the main compartment that accumulates tTG on its route to the cell surface.

Then, we focused on the vesicular and target soluble NSF attachment protein receptors (v-SNAREs and t-SNAREs) that drive fusion of recycling endosomes with the plasma membrane. VAMP3 and SNAP23 SNAREs are involved in this process in non-neuronal cells and are required for recycling of β1 integrins from the PNRC to the surface [46]. Expression of *Tetanus* neurotoxin (TeTx-LC) which inactivates VAMP3 [47], or dominant negative δc9-SNAP23 mutant [46] in NIH3T3-tTG fibroblasts, was followed by induction of tTG synthesis and analysis of its surface levels (Fig. 4B and 4C). VAMP3 cleavage or interference with SNAP23 function both reduced the rate of tTG secretion. Thus, these SNAREs are involved in the fusion of tTG-bearing endosomes with the plasma membrane.

We also used shRNA-based approach to probe the role of regulatory proteins that control endocytic trafficking pathways, in the externalization of tTG (Fig. 4D). While down-regulation of Rab4A/B, Arf6, Rab5A and Rab22A in NIH3T3-tTG fibroblasts did not affect tTG secretion, depletion of Rab11A, and even more so, of Rab11B, inhibited tTG externalization. Transient expression of the S25N-Rab11A or S25N-Rab11B dominant negative mutants [48] in these cells showed a reduction in surface tTG levels in the transfectants and confirmed the involvement of Rab11A and Rab11B in the process of tTG secretion (Fig. 4E). Hence, Rab11A/B GTPases that control endosomal trafficking from the PNRC to the cell surface [49], are involved in tTG externalization.

### tTG Interacts with Internalized β1 Integrins in the PNRC on Route to Secretion and is Delivered to the Cell Surface as a Complex with Recycled β1 Integrins

**Figure 5:**
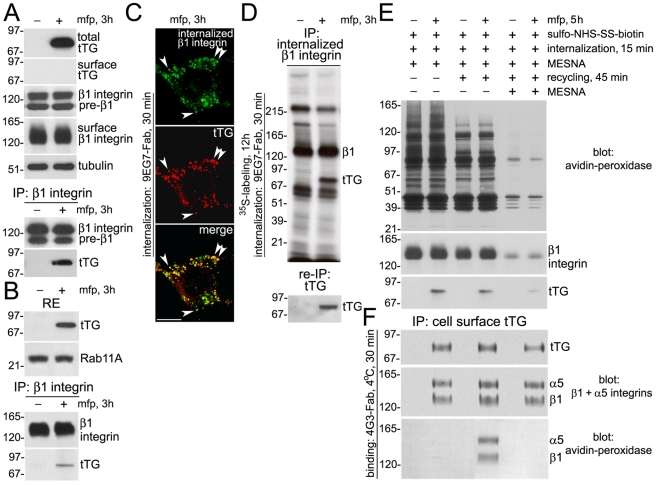
tTG Binds to β1 Integrins Undergoing the Recycling Process Within the Perinuclear Recycling Endosomes. NIH3T3-tTG fibroblasts were induced to synthesize tTG with mifepristone for 3 h (A–D) or 5 h (E,F). (A) tTG forms complexes with β1 integrins prior to secretion. The total levels of tTG, β1 integrins and tubulin were determined by immunoblotting. Cell surface levels of tTG and β1 integrins were defined by labeling cells with sulpho-NHS-LC-biotin, isolation of cell surface proteins on Neutravidin-Agarose, and immunoblotting. β1 integrins were immunoprecipitated from total cell lysates and the resulting immune complexes were probed for β1 integrin and tTG. (B) Recycling endosomes contain the β1 integrin-tTG complexes. Recycling endosomes (RE) were isolated from crude membrane fraction using magnetic beads coated with antibody to Rab11. The levels of tTG and Rab11A in the RE were defined by immunoblotting. β1 integrins were immuno-precipitated from the RE lysates and the immune complexes were probed for β1 integrin and tTG. (C) tTG colocalizes with internalized β1 integrins in perinuclear recycling endosomes. Antibody uptake experiment was performed with surface-bound 9EG7-Fab against the β1 integrin subunit. Digitonin-extracted cells were double-stained for tTG and internalized β1 integrins. Arrows mark colocalization of internalized β1 integrin-9EG7-Fab complexes with tTG on the PNRC vesicles. Immunofluorescence was analyzed by conventional microscopy. Scale bar - 10 µm. (D) The *de novo* synthesized tTG binds to internalized β1 integrins. tTG synthesis was induced 3 h before the end of metabolic labeling with ^35^S-Translabel. Antibody uptake experiment was performed with surface-bound 9EG7-Fab against the β1 integrin subunit. Internalized ^35^S-labeled β1 integrins and associated proteins were analyzed by immunoprecipitation, SDS-PAGE and fluorography. The association of tTG with internalized β1 integrins was confirmed by its reprecipitation from the β1 integrin immune complexes. (E) tTG is associated with the fraction of recycled proteins. Surface labeling and generation of the fractions of internalized proteins, (*two left lanes*), recycled proteins (*two middle lanes*), and internalized proteins retained intracellularly after the recycling (*two right lanes*), were performed as in [44], (see also Materials and Methods). After cell lysis, biotinylated and unlabeled associated proteins in these fractions were isolated on neutravidin-Agarose and β1 integrins and tTG were detected by SDS-PAGE and immunoblotting. (F) Externalized tTG is associated with the recycled α5β1 integrin. The fractions of internalized proteins, recycled proteins, and internalized proteins retained intracellularly after the recycling, were obtained as in (E). Following surface labeling and incubations, 4G3-Fab against tTG was bound to the cell surfaces at 4°C. Surface tTG and associated proteins were immunoprecipitated from cell lysates and the immune complexes were probed by immunoblotting for α5 and β1 integrins and for biotinylated (cell surface-derived) proteins by blotting with neutravidin-peroxidase. Shown in (A,B,D-F) are representative of three independent experiments.

Since the *de novo* synthesized tTG forms complexes with its cell surface binding partners β1 integrins prior to externalization [20], and recycling of β1 integrins from the PRNC to the cell surface is Rab11-dependent [48], [49], we sought to determine whether the two proteins interact inside this compartment prior to tTG externalization (Fig. 5). Using co-immunoprecipitation analysis after induction of tTG synthesis for 3 h in the NIH3T3-tTG fibroblasts, we confirmed our earlier observations with human erythroleukemia (HEL) cells [20] that this protein is associated with β1 integrins prior to externalization (Fig. 5A). This association occurs inside the PNRC, as *de novo* synthesized tTG was detected in the β1 integrin immune complexes isolated from the Rab11-positive recycling endosomes (Fig. 5B) and was partilally co-localized in the perinluclear vesicles with the β1 integrins internalized from the surface of NIH3T3-tTG fibroblasts (Fig. 5C). Finally, we confirmed the binding of *de novo* synthesized tTG to the internalized β1 integrins in these cells by co-immunoprecipitation analysis after metabolic labeling (Fig. 5D).

Next, we set to examine whether tTG is exported in these cells as a complex with intracellular β1 integrins recycled back to the cell surface (Fig. 5E,F). Cell surface proteins were labeled with reducible sulfo-NHS-SS-biotin 3 h after induction of tTG synthesis, allowed to undergo endocytosis for 15 min, and the remaining surface biotin label was stripped with the reducing agent MESNA (Fig. 5E, [44]). The fractions of internalized proteins, the ones recycled back to the surface for additional 45 min after the MESNA treatment, and the ones retained intracellularly after the recycling, were all isolated on neutravidin-Agarose beads. In agreement with previous report [44], the majority of internalized proteins, including >80% of β1 integrins, recycled back to the surface of the NIH3T3-tTG fibroblasts under these conditions (Fig. 5E). Notably, immunoblotting revealed an association of *de novo* synthesized tTG with both the internalized and recycled proteins in these cells (Fig. 5E). To identify the binding partners for secreted tTG in these cellular fractions, cell surface-bound Fab fragment of mAb 4G3 [21] was used to pull-down externalized tTG together with associated proteins (Fig. 5F). Probing the resulting tTG immune complexes for α5 and β1 integrins by immunoblotting and the cell surface-derived proteins by blotting with neutravidin-peroxidase revealed an association of externalized tTG with the recycled α5β1 integrin. Therefore, *de novo* synthesized tTG interacts with internalized β1 integrins in the PNRC on its route to secretion and is delivered to the cell surface as a complex with recycled β1 integrins.

### Interaction with Phosphoinositides Is Required for Targeting Cytoplasmic tTG to Endosomal Membranes and Externalization

**Figure 6:**
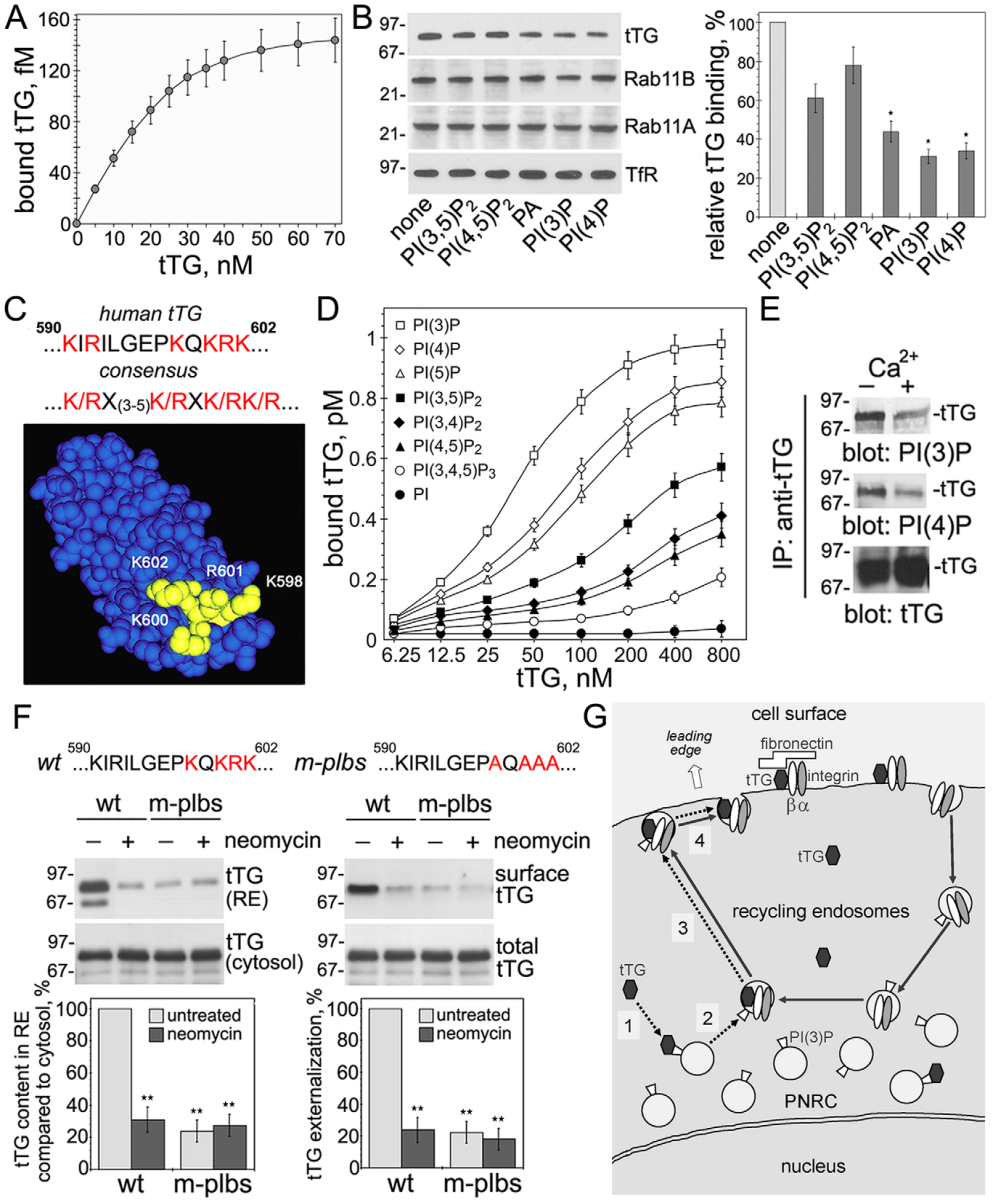
Blocking Phosphoinositide Binding Inhibits the Recruitment of Cytoplasmic tTG to Endosomal Membranes and Impairs Its Externalization. (A) Binding of tTG to recycling endosomes. The amounts of purified ^125^I-tTG bound at 4°C to recycling endosomes immunoisolated from NIH3T3 cells lacking tTG were determined in a gamma counter with all measurements performed in triplicates. Shown are means ± SEM for three independent experiments. (B) The role of phosphoinositides in the tTG interaction with recycling endosomes. Binding of 50 nM purified tTG to recycling endosomes was tested in the presence of 100 µM free phosphoinositides. Bound tTG and endosomal Rab11A/Rab11B and transferrin receptor (TfR) were detected by immuno-blotting. The relative amounts of vesicle-bound tTG were quantified and compared to that in the sample without phosphoinositides in four independent experiments. Bars depict means ± SEM, *p<0.05. (C) The putative phospholipid-binding sequence and site within the fourth domain of tTG. (D) Comparative analysis of tTG binding to phosphoinositides. ^125^I-tTG binding to synthetic liposomes (PolyPIPosomes™) containing indicated phiosphoinositides was determined in a gamma counter with all measurements performed in triplicates. Shown are means ± SEM for three independent experiments. (E) Association of tTG with phosphoinositides in cells. tTG was immunoprecipitated from extracts of WI-38 fibroblasts and the immune complexes were probed by immunoblotting for PI(3)P or PI(4)P. (F) The phospholipid-binding site in tTG is required for membrane targeting and externalization of the protein. K598A,K600A,R601A,K602A mutations (m-plbs) were generated within the phospholipid-binding site of tTG. NIH3T3 fibroblasts transfected with wild type tTG (wt) or its mutant deficient in phosphoinositide binding (m-plbs) were left untreated or treated with 10 mM neomycin for 24 h before induction of these proteins for 4 h. The contents of wt and m-plbs tTG in the recycling endosomes and cytosol were defined by immunoblotting (*left panels*). Cell surface and total levels of wt and m-plbs tTG were defined by surface biotinylation, isolation of surface protein fractions, and immunoblotting (*right panels*). The relative contents of wt and m-plbs tTG in the recycling endosomes and on the cell surface were quantified and compared to those for untreated cells expressing wt protein. Shown are representative of three independent experiments. Bars depict means ± SEM, **p<0.005. See also Figure S5. (G) A proposed mechanism of unconventional tTG secretion. The *de novo* synthesized intracellular (cytoplasmic) tTG (*hexagons*) does not follow the classical, ER/Golgi-dependent secretion pathway, but is exported via a four-step unconventional secretion process mediated by recycling endosomes (*dotted arrows*). *Solid arrows* mark the major intracellular recycling route through the PNRC utilized by β1 integrins. See further comments in the text.

We hypothesized that phospholipids of endosomal membranes might be involved in targeting tTG to the PNRC and measured the binding of tTG to these organelles after their immunoisolation from NIH3T3 cells lacking this protein (Fig. 6A). The recombinant tTG exhibited a specific, dose-dependent and saturable binding to the recycling endosomes with the *K*_d_ ∼20 nM. Preincubation of tTG with 100 µM purified phosphatidyl-inositol (3,5)-diphosphate [PI(3,5)P_2_], phosphatidylinositol (3,4)-diphosphate [PI(4,5)P_2_], phosphatidic acid [PA], phosphatidylinositol (3)-phosphate [PI(3)P] or phosphatidylinositol (4)-phosphate [PI(4)P] diminished its binding to the vesicles with the latter two phosphoinositides having the strongest inhibitory effect, yet it did not affect the relative content of Rab11A/Rab11B on these vesicles (Fig. 6B). Thus, binding to membrane phospholipids is involved in the association of cytoplasmic tTG with transport vesicles.

In search for molecular motifs involved in the tTG binding to phosphoinositides, we identified the sequence …(590)**K**I**R**ILGEP**K**Q**RKK**(602)… that fits the consensus **K/R**X(3–5)**K/R**X**K/RK/R** for phospholipid binding (Fig. 6C, [50], [51]) and is not shared by other members of the transglutaminase family [18]. The residues K598, K600, R601 and K602 form a positively charged cluster on the surface of the fourth domain (*marked in yellow*). *In vitro* binding assays with recombinant tTG and immobilized membrane lipids (Fig. S5A) or phosphoinositides (Fig. S5B) showed its interaction with PA, phosphatidylserine (PS), PI(4)P, PI(3)P, phosphatidylinositol (5)-phosphate [PI(5)P], PI(3,5)P_2_, PI(4,5)P_2_, phosphatidyl-inositol (3,4)-diphosphate [PI(3,4)P_2_], and non-phospholipid membrane compounds cardiolipin and sulfatide. In all the cases except that of sulfatide, the interaction was inhibited by Ca^2+^. Yet, only a weak interaction with phosphatidylinositol (3,4,5)-triphosphate [PI(3,4,5)P_3_], and no binding to phosphoinositol (PI), phosphatidylcholine (PC), phosphatidylethanolamine (PE), cholesterol, and sphyngomyelin was observed. A quantitative *in vitro* analysis of tTG interaction with synthetic phosphoinositide-containing liposomes revealed that it binds monophosphates PI(3)P, PI(4)P and PI(5)P with highest affinity, while diphosphates PI(3,4)P_2_, PI(3,5)P_2_, and PI(4,5)P_2_ exhibit weaker interaction and triphosphate PI(3,4,5)P_3_ only negligibly binds to tTG (Fig. 6D). This order of interactions is reverse to typical for most phospholipid-binding proteins [50], [51] and is due to negatively charged E596 residue adjacent to the positively charged cluster. Importantly, immunoprecipitation from WI-38 fibroblasts showed a tight tTG binding to PI(3)P and PI(4)P and inhibition of this interaction by Ca^2+^ (Fig. 6E), whereas the recombinant tTG from *E. coli* had no bound phospholipids (Fig. S4C). Thus, the phospholipid-binding site in this protein allows it to interact with a wide range of membrane phosphoinositides.

Finally, we examined the role of phospholipid binding in the interaction of cytoplasmic tTG with transport vesicles and its trafficking to the cell surface (Fig. 6F). We used neomycin, a drug that blocks the interaction of proteins with phosphoinositides [9] and generated the tTG mutant K598A,K600A,R601A,K602A (m-plbs) with altered phospholipid-binding site, which failed to bind PI(3)P and PI(4)P upon expression in NIH3T3 fibroblasts (Fig. S4D). Both neomycin treatment and mutation of the phospholipid-binding site reduced the association of tTG with recycling endosomes and inhibited its secretion. We concluded that the interaction with phosphoinositides is required for recruitment of cytoplasmic tTG to recycling endosomes and subsequent externalization.

## Discussion

In this study we begin to delineate the unconventional mechanism of tTG secretion. We find that externalization of tTG to the cell surface does not require the ER/Golgi function, but follows a non-classical vesicle-mediated pathway based on its recruitment to the membranes of PNRC and utilizing the long endosomal recycling loop for secretion. The initial recruitment of cytoplasmic tTG to the endosomal membranes depends on phosphoinositide binding, and membrane-bound tTG is delivered inside the perinuclear endosomes prior to externalization. Finally, regulatory Rab11A/Rab11B GTPases that control outbound trafficking of the tTG-bearing recycling endosomes, and VAMP3 and SNAP23 SNAREs that mediate endosome to plasma membrane fusion, are all involved in tTG secretion. Hence, this work provides a novel example of the use of endosomal recycling pathway for non-classical externalization of cytoplasmic secretory protein.

Based on the observations presented here, we propose a pathway of constitutive tTG secretion which is likely common for many cell types that express this protein (Fig. 6G). It includes: {1} phospholipid-dependent binding of cytoplasmic tTG to the PNRC vesicles; {2} delivery of the membrane-bound tTG inside these transport vesicles; {3} anterograde movement of these vesicles; and {4} their fusion with the plasma membrane which exposes intravesicular tTG to the extracellular space. Several features of this pathway distinguish it from other reported or proposed mechanisms of unconventional secretion pathways for cytoplasmic proteins (for detailed review see [3]–[7]). The requirement for endosomal targeting sets it apart from vesicle-independent non-classical secretion pathways described for FGF1 and FGF2 [5]–[7]. The lack of lysosomal targeting of tTG and insensitivity of its trafficking to inhibitors of lysosomal function indicates that the tTG secretion route does not involve protein sequestration by secretory lysosomes as shown for inflammation-induced IL-1β release by macrophages [10], [11]. Although in macrophages various inflammatory cytokines increase surface tTG level [21], this occurs via transcriptional upregulation rather than induction of its trafficking and externalization [18]. Moreover, in all the reported cases cytoplasmic tTG undergoes a constitutive secretion in diverse types of cells [18], [19]. Thus, unlike most routes of unconventional secretion, the default tTG secretion pathway is designed to operate in a wide variety rather than in a selected cell type, as is the case for FGF2 in fibroblasts [6], [9] or IL-1β in macrophages [10]-[13]. Likewise, the delivery of tTG onto the cell surface precedes its extracellular appearance, indicating that the tTG secretion pathway does not involve shedding of microvesicles at the extracellular side of the plasma membrane and exosome release [6], [12], [13].

At the moment, the mechanism(s) of tTG delivery inside the PNRC vesicles remain unknown. While we do not rule out a potential role for transmembrane transporters [6], [8], [10] in shuttling the endomembrane-bound tTG inside the PNRC, our biochemical experiments with isolated endosomes and the dominant negative NSF mutant, combined with electron microscopy observations, favor an involvement of membrane fusion events and/or endosomal budding in the delivery of endomembrane-bound tTG inside the transport vesicles. Also, one can not exclude an involvement of Rab11-containing amphisomes and/or autophagosomes [8], [14], [15], [53] and their fusion with recycling endosomes, in the process of tTG secretion. Furthermore, the localization of tTG on intralumenal vesicles of multivesicular endosomes reported in this study may result from the ESCRT-independent inward budding and scission of the limiting endosomal membrane during the formation of multivesicular endosomes [52]. Finally, our data implicate membrane fusion events in the fusion of the tTG-bearing recycling trasport vesicles with the plasma membrane at the final stage of tTG delivery onto the cell surface.

In addition to some unique features of this unconventional secretion pathway, the constitutive route of tTG externalization shares several general principles utilized in unconventional secretion of other cytoplasmic proteins. For cytoplasmic tTG, targeting cargo protein to endosomal membranes occurs via its interaction with a subset of phosphoinsitides, like in the case of FGF2 [9], although spatial specificity of membrane docking sites (inner leaflet of plasma membrane versus endosomal membrane) are different. The preferential binding of tTG to PI(3)P fits well the phospholipid composition of endosomal membranes that are enriched in this phosphoinositide [49]. Further, the interaction of cytoplasmic tTG with intracellular transport vesicles may represent a two-step process with its initial tethering to endosomal phospholipids and subsequent tight binding to endomembrane protein(s) acting as tTG receptor(s). A search for such tTG-binding partner(s) on the endosomal membranes is currently underway. Finally, Ca^2+^ serves as a common regulator of this and other non-classical secretion pathways by promoting vesicular trafficking and/or membrane fusion events [54].

The emerging relationship of this unconventional trafficking pathway to the general recycling routes of integrins has important functional implications (Fig. 6G). Several features of tTG secretion including its dependence on Rab11 function and VAMP3- and SNAP23-mediated endosome to plasma membrane fusion coincide with those governing integrin recycling [44], suggesting that tTG is exported inside the same vesicles that contain integrins undergoing the recycling process. We identified β1 and β3 integrins as the principal binding partners for tTG on the cell surface, and showed a major role of the integrin-tTG complexes in cell-ECM interactions and outside-in signaling [20], [21], [55]. Moreover, our previous studies indicated that tTG binds β1 integrin within 30–60 min after onset of biosynthesis [20], but the lack of tTG in the ER/Golgi left unresolved the issue of cellular compartment in which these complexes are formed. The targeting of cytoplasmic tTG to the PNRC provides a novel explanation for these earlier findings. Both β1 and β3 integrins undergo endocytosis with the former utilizing the long, PNRC-mediated, and the latter - the short recycling routes [44]. While low levels of αvβ3 integrin in the NIH3T3-tTG fibroblasts [55], [56] precluded analysis of its intracellular interaction with tTG, we identified the internalized α5β1 integrin as a key binding partner for tTG inside the PNRC vesicles and showed that *de novo* synthesized tTG is externalized as a complex with this integrin via the long recycling route. We envision that targeted delivery of intracellular adhesive/signaling integrin-tTG complexes to lamellipodia should strengthen adhesion to the ECM at the leading edge of migrating cell and contribute to directionality of cell migration. This hypothesis will be tested in our future work.

## Materials and Methods

### Reagents and Antibodies

Unless stated otherwise, all chemicals were obtained from Sigma-Aldrich. NEM was obtained from EMD Biosciences. ^35^S-Translabel was obtained from MP Biomedicals. Sulfo-NHS-LC-biotin, Sulfo-NHS-SS-biotin, neutravidin-Agarose, neutravidin-peroxidase, Protein G-Agarose, PVDF membranes, secondary peroxidase-labeled IgGs, and ECL reagents were from Pierce Biotechnology. Mifepristone was from Invitrogen (#H11001). 6 nm goat anti-mouse IgG gold conjugate and 10 nm goat anti-rabbit IgG gold conjugate (Aurion) were from Electron Microscopy Sciences. Dynal® Dynabeads coupled with anti-mouse IgG, anti-rabbit IgG, or streptavidin for isolation of biotinylated plasma membrane vesicles, were from Invitrogen. PE-labeled secondary antibodies were from BD Biosciences. Purified recombinant His-tagged human tTG was obtained from Zedira. Membrane lipid strips (#P6002); PIP strips (#P6001), PolyPIPosomes (#YP000; #YP003; #YP004; #YP005; #YP034; #YP035; #YP045; #YP039), and purified synthetic phosphoinositides were from Echelon Biosciences. Membrane styryl dye FM4-64FX and secondary IgGs conjugated with Alexa Fluor 488 and Alexa Fluor 594 were from Molecular Probes/Invitrogen. mAbs CUB7402 and TG100 against tTG were from LabVision/Neomarkers. mAb 4G3 against human tTG was characterized earlier [21]. Antibody to 6xHis tag was from Genscript. Rat anti-mouse/human β1 integrin, clone 9EG7, was from BD Biosciences, #553715. Rabbit antibody to the cytoplasmic domain of β1 integrin was from Millipore, #AB1952. Antibody #610657 to Rab11A/Rab11B was from BD Biosciences. Antibodies to β-actin, #sc8432; β-tubulin, #sc9104; c-myc, #sc-40; Rab7, #sc10767; Rab11A, #sc25690; Rab11B, #sc26591; EEA1, #sc6414; LAMP1; #sc8098, LAMP3, sc#98658; VAMP3, sc#18208; calnexin, #sc6465; were all obtained from Santa Cruz Biotechnology. Polyclonal antibody against GM130 was described earlier (57). Rabbit polyclonal antibodies to Tsg101, ab30871, and Vps24, ab76333, were obtained from Abcam. Antibodies to PI(3)P, #Z-P003, and PI(4)P, #Z-P004, were from Echelon Biosciences. Dynasore, a cell-permeable small molecule that inhibits the GTPase activity of dynamin-1, dynamin-2, and Drp1 (58), was obtained from Tocris Biosciences (#2897).

### Cells, Constructs and Transfections

NIH3T3 fibroblasts in which expression of tTG or tTG-His/myc is based on pSwitch-pGene dual plasmid expression system (Invitrogen) were described [26], [34]. HUVECs (Invitrogen) were used between 6^th^ and 10^th^ passages. WI-38 human lung fibroblasts were from ATCC. Mutations of the K598, K600, R601 and K602 residues to alanines in tTG were introduced by PCR-based site-directed mutagenesis. wt-NSF-myc, E239Q-NSF-myc, TeTx-LC, SNAP23-myc, δc9-SNAP23-myc, GFP-wt-Rab11A/B, and GFP-S25N-Rab11A/B constructs were expressed in NIH3T3-tTG fibroblasts using transfection with Neofectin (MidAtlantic Biolabs). Transient expression of pre-designed and verified shRNAs for mouse Rab4A/B, Rab11A/B, Arf6, Rab5A, Rab22A, Tsg101, and Vps24 in pGFP-V-PS vector in these cells was achieved by their transfection with Turbofectin 8.0 (all from Origene Technologies).

### Inhibitors

The following inhibitors were used: brefeldin, 0.5 µg/ml; tunicamycin, 0.2 µg/ml; monensin, 2 µM; bafilomycin, 50 nM; cytochalasin D, 2 µg/ml; nocodazole, 0.5 µg/ml; sodium chlorate, 75 mM; and glyburide, 50 µM. To alter intracellular [Ca^2+^], BAPTA (5 µM) and Ca^2+^ ionophore (10 µM) were used. NEM (0.6 mM) was used to block ATPase activity of NSF. Most inhibitors were applied 30 min before tTG induction or start of metabolic labeling. Sodium chlorate was added 12 h before tTG induction. Neomycin, the drug that slowly penetrates plasma membrane and sequesters phosphoinositides was used for 18 h at 10 mM.

### Cell Surface Biotinylation, Biosynthetic Labeling, and Detection of Surface tTG and β1 Integrin

To define the surface levels of tTG and β1 integrin, cells were labeled for 15 min at 4°C with 0.5 mg/ml cell impermeant Sulfo-NHS-LC-biotin [26], [34]. Biotinylated proteins were isolated on Neutravidin-Agarose. Metabolic labeling with ^35^S-Translabel and quantitative immunoprecipitation of tTG and β1 integrins in the presence of detergents was reported [20], [53]. To isolate *de novo* synthesized surface tTG and β1 integrins, cells were labeled with 200 µCi/ml ^35^S-Translabel for 15 min and then chased with medium containing no radioactivity. At the end of labeling/chase, cells were surface-biotinylated and the fraction of surface proteins was obtained as above.

### Immunofluorescence Microscopy

WI-38 and NIH3T3-tTG or NIH3T3-tTG-His/myc fibroblasts [34], [39], [52] on glass coverslips were fixed with 3% formaldehyde and permeabilized with 0.1% Triton X-100, fixed/permeabilized with ice-cold methanol, or permeabilized at 4°C with 0.1% digitonin in QP buffer containing 4% PEG 40,000, 20 mM PIPES pH 6.9, 50 mM KCl, 1 mM EDTA, and then fixed with 3% formaldehyde. Cells were either labeled for tTG with mAbs CUB7402/TG100 or for exogenous His/myc tagged tTG with anti-6xHis antibody, and secondary Alexa Fluor 488-labeled IgGs, or double-labeled for tTG and polyclonal antibodies Rab11, Rab7, or LAMP1, followed by secondary IgGs conjugated with Alexa Fluor 488 and Alexa Fluor 594. To relate the intracellular tTG distribution to membrane compartments, the intracellular membranes were labeled for 10 min with 10 µg/ml styryl dye FM4-64FX 30 min before cell fixation and labeling with anti-tTG antibody. Cells were viewed and photographed with 100x objective using a Nikon Eclipse E800 microscope (Nikon) and SPOT RT digital camera. Images were acquired and digitally merged with Advance Spot software (Diagnostic Instruments). Alternatively, stained cells were examined with 100x objective using Zeiss/Bio-Rad 2000 confocal microscope. Images were acquired and digitally merged with Volocity software (Improvision).

### Immunoelectron Microscopy

For immunoEM analysis, cells were fixed in 4% paraformaldehyde, 0.1 M PIPES buffer (pH 7.35), scraped off the tissue culture vessel, washed, pelleted and enrobed in 2.5% low melting temperature agarose. Agarose blocks containing cells were trimmed into ∼1 mm^3^ size, washed and dehydrated by gradually lowering temperature from 4°C to −20°C in increasing concentration of ethanol, the infiltrated and embedded in unicryl at −20°C during 24–48 h. Ultrathin sections were cut on Leica UC6 microtome (Leica Microsystems) and collected onto formvar coated nickel grids. Grids were inverted section-side facing down onto a drop of blocking solution containing 1% BSA, 1% fish gelatin, 0.01 M glycine in PBS, pH 7.4 for 10 min and transferred on a droplet of primary antibody diluted in blocking solution for 30 min at room temperature. Then, the grids were washed and labeled with 6 mm gold and 10 nm-gold conjugated secondary antibody the same way followed by washing. Finally, grids were fixed with 2% glutaraldehyde in PBS for 5 min, rinsed with water, air dried and examined using FEI Technai T12 transmission electron microscope at 80 kV. Images were acquired with AMT-XR611 digital camera (Advanced Microscopy Techniques) by using AMTV600 software.

### Inactivation of Recycling Endosomes and Lysosomes

The ablation of recycling endosomes was performed as in [43]. NIH3T3-tTG fibroblasts were incubated with 0.02 mg/ml Tfn-HRP (Jackson Immunoresearch Laboratories, #015-030-050) in media for 30, washed once in serum-free DMEM, and incubated another 15 min and washed twice in ice-cold PBS. Surface-bound Tfn-HRP was removed by two 5-min washes in 0.15 M NaCl and 20 mM citric acid, pH 5.0. Then, cells were washed with ice-cold PBS, pH 7.4, and resuspended in PBS containing 0.1 mg/ml DAB (Sigma). H_2_O_2_ was added to a final concentration 0.03% to the inactivation sample; PBS was added to the control set. Cells were incubated on ice for 45 min in the dark and the reaction was stopped by washing cells twice in PBS/BSA 1%. To ablate lysosomes, a similar procedure was performed with 0.5 mg/ml free HRP, which accumulates at lysosomal compartments. At the end of endosomal/lysiosomal ablation, tTG synthesis in the cells was induced with mifepristone and tTG surface levels were determined as reported [26], [34].

### Subcellular Fractionation, Immunoisolation of Organelles and Proteinase Protection Assays

Subcellular fractionation in the absence of detergents was performed as described by Andrei et al. [10]. The fraction of light and medium membranes, lacking nuclei and mitochondria and containing endosomes, lysosomes, ER, Golgi, and plasma membrane vesicles was further used for immuno-isolaion of organelles. Primary antibodies directed against the markers of these organelles were bound to Dynabeads™ M-280 pre-coated with sheep anti-rabbit or anti-mouse IgGs (Dynal) according to the manufacturer's instructions. The crude fraction of light and medium membranes (P2 fraction in Andrei et al. [10]) was incubated with primary antibody-coated magnetic beads for 5 h with continuous slow rotation at 4°C. Bound immunocomplexes were captured using a magnetic device, washed four times in 20 mM Tris-HCl (pH 7.4) and 150 mM NaCl, and analyzed for tTG content by immunoblotting together with an aliquot of the input. To isolate plasma membrane vesicles, NIH3T3-tTG fibroblasts were incubated with 100 µg/ml biotin-labeled Concanavalin A (Sigma) to bind it to the outside leaflet of the plasma membrane. Then, streptavidin-coated Dynabeads™ (Dynal) were used for affinity isolation of plasma membrane vesicles (59). Proteinase protection experiments with immunoisolated recycling endosomes were performed by treatment of vesicles with 0.1 mg/ml proteinase K with or without 0.1% Triton X-100, sonication for 3×10 sec with pistol-type sonicator, or with 1 M KCl, for 30 min at 4°C.

### Binding of tTG to Recycling Endosomes and Membrane Translocation Experiments

The binding of 0-80 nM [^125^I]tTG (sp. act. 1.2×10^6^ cpm/µg) to recycling endosomal vesicles was studied at 4°C in the buffer containing 10 mM TrisCl, pH 6.8, 140 mM NaCl, 5 mM EDTA, 1 mM EGTA. Preincubation with 100x excess of unlabeled tTG was used to determine and subtract non-specific binding. After 30 min incubation, vesicles were extensively washed by centrifugation, and bound radioactivity was quantified in a gamma counter. In other experiments, binding of 50 nM unlabeled tTG to the vesicles was studied after preincubation with 100 µM purified phosphoinositides. The tTG bound to vesicles was detected and quantified by SDS-PAGE and immunoblotting and normalized to the levels of tubulin and Rab11A/Rab11B in these vesicles.

To study the inward translocation of vesicle-bound tTG, the recycling endosomes and the cytosol were isolated from the NIH3T3 cells lacking tTG. The vesicles were incubated for 1 h at 4°C with exogenous tTG, washed, warmed to 37°C for 1 h in the presence or absence of 5 mM Mg^2+^/2 mM ATP and cytosol, and then treated with proteinase K with or without Triton X-100 [10]. The state of vesicle-bound tTG was defined by SDS-PAGE and immunoblotting.

### Isolation of the Fractions of Internalized and Recycled Proteins

The fraction of biotinylated internalized proteins in NIH3T3-tTG fibroblasts was obtained as in [44] by labeling cells with SH-cleavable sulfo-NHS-SS-biotin 3 h after induction of tTG synthesis, internalization of biotinylated surface proteins for 15 min at 37°C, and stripping the remaining surface biotin with MESNA. The fraction of biotinylated recycled proteins was isolated similarly upon subsequent recycling of internalized proteins to the cell surface for 45 min at 37°C. The fraction of biotinylated internalized proteins retained intracellularly after the recycling was obtained following second MESNA stripping. After cell lysis, biotinylated and unlabeled associated proteins in these fractions were isolated on neutravidin-Agarose.

### Antibody Uptake Experiments

The binding of Fab fragments of mAb 4G3 against tTG and mAb 9EG7 against β1 integrins to the cell surfaces and uptake experiments were performed as reported earlier [34].

### *In vitro* Binding Experiments with tTG and Phosphoinositides

To determine the interactions of tTG with phospholpid arrays *in vitro*, membrane lipid and PIP strips were blocked for 1 h with 1% obalbumin in 50 mM TrisCl pH 7.5; 150 mM NaCl, 0.1% Tween 20. Purified tTG (0.5 µg/ml) was incubated for 1 h at 25°C with the membranes in the same buffer containing either 1 mM EDTA or 5 mM CaCl_2_. The bound protein was detected by immunoblotting for tTG, followed by secondary peroxidase-labeled IgGs and ECL development.

To define the binding of tTG to synthetic liposomes containing 5% (w/w) individual phosphoinositides (PolyPIPosomes™, Echelon Biosciences), 25 µl liposome preparations were incubated on a rotator with 0-800 nM purified recombinant [^125^I]tTG in 10 mM TrisCl pH 6.8, 140 mM NaCl, 5 mM EDTA, 1 mM EGTA for 30 min at 4°C. Preincubation with 100x excess of unlabeled tTG was used to determine and subtract non-specific binding. At the end of incubation, liposomes were washed 5 times with the same buffer by centrifugation, and the amounts of bound tTG were quantified in a gamma counter.

### Flow Cytometry

Live non-permeabilized NIH3T3-tTG trasfectants expressing GFP fusion constructs with shRNAs for Rab4A/4B, Rab11A/11B, Arf6, Rab5A and Rab22A; as well as with wt-Rab11A, S25N-Rab11A, wt-Rab11B, and S25N-Rab11B, were stained for cell surface tTG at 4°C with a mixture of mAbs TG100, CUB7402 and 4G3 and phycoerythrin- (PE)-labeled secondary antibody as described [26], [34], [52]. At the end of the procedure, the cells were washed and fixed with 0.5% formaldehyde. Two color flow cytometry of cells was performed using FACSCanto™ instrument and FACSDiva software (BD Biosciences). The results were analyzed for gated intact cells using FCS3 Express software (DeNovo).

### Densitometry and Statistical Analysis

Peroxidase-conjugated secondary antibodies and enhanced chemiluminescence (ECL) was used for signal detection. The signals for protein bands were quantified with NIH Image 1.63f software. Statistical significances were determined using unpaired, two-tailed Student's t tests assuming equal variances and an alpha level of 0.05. Differences were considered significant if the p value was <0.05.

## Supporting Information

Figure S1 **Dynamic equilibrium between externalization and endocytic removal of tTG from the cell surface.** NIH3T3-tTG fibroblasts were left untreated or were treated for 30 min with 200 μM dynamin inhibitor dynasore before induction of tTG synthesis with mifepristone. The surface levels of tTG and β1 integrin were defined after cell surface biotinylation and isolation of surface proteins (see Materials and Methods) by immunoblotting of cell surface protein fraction. The relative amounts of tTG and β1 integrin on the surface of dynasore-treated NIH3T3-tTG fibroblasts were compared to those in untreated cells. Shown is representative of three independent experiments. Bars depict means ± SEM, *p<0.05. The total tTG and actin levels were defined by direct immunoblotting. Note that dynasore increases surface levels of tTG and β1 integrin. However, tTG internalization from the cell surface affects its levels during the late, but not the early phase of secretion. Related to Figure 1.
(TIF)

Figure S2 **Heat shock and Cu^2+^ chelator do not affect tTG externalization.** NIH3T3-tTG fibroblasts were treated for 18 h with 0-0.2 mM Cu^2+^ chelator ammonium tetrathiomolybdate (TTM) before inducton of tTG synthesis for 4 h. During the last hour of tTG synthesis, cells were left at 37°C or switched to 42°C. The surface tTG levels were defined after cell surface biotinylation and isolation of surface proteins (see Materials and Methods) by immunoblotting of cell surface protein fraction. The relative tTG levels on the surface of NIH3T3-tTG fibroblasts were compared with those in untreated cells at 37°C. Shown is representative of three independent experiments. Bars depict mean values ± SEM. The total tTG and tubulin levels were defined by direct immunoblotting. Note that heat shock and alteration of cellular Cu^2+^ levels, which affect the non-classical secretion of FGF1 [36], do not alter tTG externalization. Related to Figure 2.
(TIF)

Figure S3 **Intracellular localization of tTG in fibroblasts.** (A,B) NIH3T3-tTG-His/Myc fibroblasts [39] were induced to synthesize His/myc-tagged tTG for 24 h (A) or indicated time (B). Cells were either fixed and permeabilized with formaldehyde and Triton X-100 (A), or extracted with digitonin before fixation (B), and then stained for tTG with antibody to 6xHis tag. Note tTG localization of in focal adhesions (arrowheads, (A)) and in perinuclear vesicles (asterisks, (B)). Immunofluorescence was analyzed by conventional microscopy. (C,D) NIH3T3-tTG fibroblasts were induced to synthesize tTG for 3 h. (C) Digitonin-extracted cells were double-stained for tTG and the late endosomal marker Rab7, or lysosomal marker Lamp1. Inserts show magnified perinuclear areas. Note a general lack of tTG co-localization with late endosomes and lysosomes. Immunofluorescence was analyzed by laser confocal microscopy. Bars - 10 μm. (D) Immunoelectron microscopic localization of tTG in NIH3T3-tTG fibroblasts. Double labeling of thin sections was performed for tTG (6 nm gold, arrowheads) and Rab11 (10 nm gold, arrows). *NM* - nuclear membrane; *MVE* - multivesicular endosome; *ILV* - intraluminal vesicle, also is shown as insert at higher magnification. Note the localization of tTG inside multivescular endosome on intraluminal vesicle. Related to Figure 3.
(TIF)

Figure S4 **ESCRT function is not involved in tTG secretion.** Depletion of Tsg101 and Vps24, the components of ESCRT-I and ESCRT-III complexes, respectively, was achieved by simultaneous transfection of shRNAs for these proteins into NIH3T3-tTG fibroblasts. tTG synthesis in these and control transfectants expressing scrambled shRNAs was induced for 4 h prior to cell surface biotinylation and isolation of surface proteins. Cell surface tTG levels and total levels of TSG101, Vps24, tTG, and tubulin were defined by immunoblotting. The relative surface level of tTG in the TSG101-, Vps24-depleted cells was compared to that in control transfectants expressing scrambled shRNAs. Shown is a representative of three independent experiments. Bars show means ± SEM. Related to Figure 3.
(TIF)

Figure S5 **The interaction of tTG with phospholipids** ***in vitro*** **and in cells.** (A,B) Interaction of tTG with phospholipids *in vitro*. Specificity and Ca^2+^ dependence of the interaction of tTG with membrane lipids (A) and phospholipids (B) *in vitro* was studied with membrane arrays (Echelon Biosciences). Bound tTG was detected by immunoblotting. (C,D) Interaction of tTG with phospholipids in cells. (C) tTG was immunoprecipitated from extracts of WI-38 fibroblasts. The resulting immune complexes and recombinant tTG purified from *E. coli* (Zedira) were analyzed by SDS-PAGE and immunoblotting with antibodies against PI(3)P and PI(4)P. Only the endogenous protein from fibroblasts, but not the recombinant tTG binds phosphoinositides. (D) Mutation of the presumed phospholipid-binding site interferes with the tTG-phosphoinositide association in fibroblasts. Wild type (wt) and K598A,K600A,R601A,K602A (m-plbs) mutant were expressed in NIH3T3 fibroblasts, then immunoprecipitated from cell extracts and tested for bound phospholipids by immunoblotting with antibodies against PI(3)P or PI(4)P. Shown in (A-D) are representative of three independent experiments. Related to Figure 6.
(TIF)
