# Supplementary figures and images for: Unconventional Secretion of Tissue Transglutaminase Involves Phospholipid-Dependent Delivery into Recycling Endosomes
Source: PLoS One. 2011 Apr 27;6(4):e19414. doi: 10.1371/journal.pone.0019414 (PMC3083433; doi:10.1371/journal.pone.0019414)

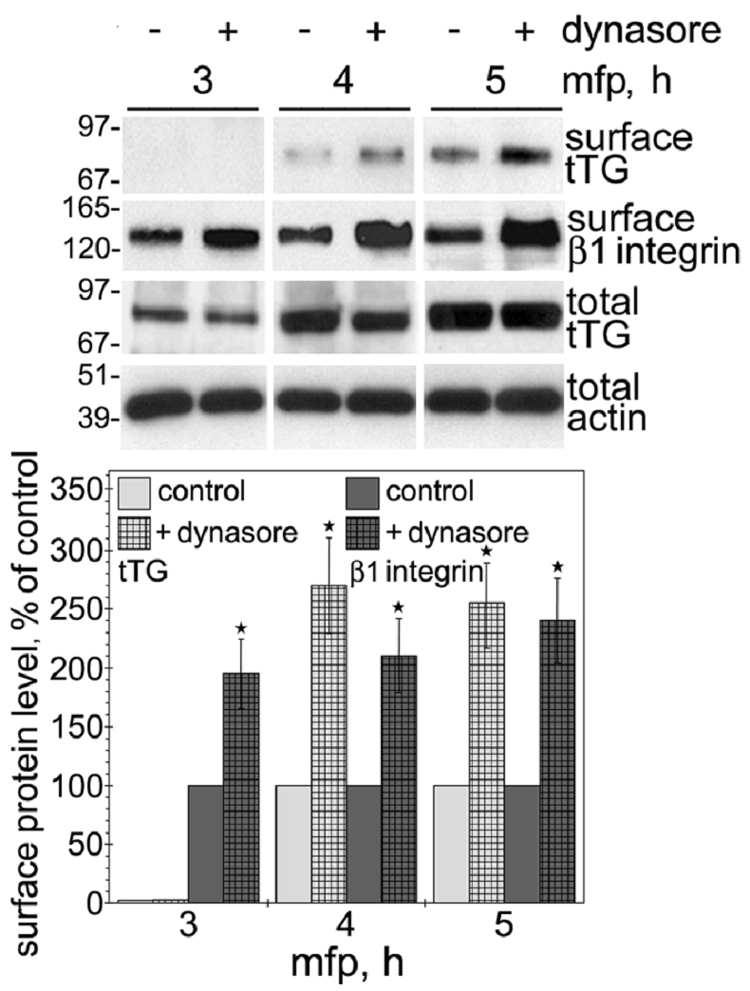

Supplement: Figure S1 — Dynamic equilibrium between externalization and endocytic removal of tTG from the cell surface. NIH3T3-tTG fibroblasts were left untreated or were treated for 30 min with 200 μM dynamin inhibitor dynasore before induction of tTG synthesis with mifepristone. The surface levels of tTG and β1 integrin were defined after cell surface biotinylation and isolation of surface proteins (see Materials and Methods) by immunoblotting of cell surface protein fraction. The relative amounts of tTG and β1 integrin on the surface of dynasore-treated NIH3T3-tTG fibroblasts were compared to those in untreated cells. Shown is representative of three independent experiments. Bars depict means ± SEM, *p<0.05. The total tTG and actin levels were defined by direct immunoblotting. Note that dynasore increases surface levels of tTG and β1 integrin. However, tTG internalization from the cell surface affects its levels during the late, but not the early phase of secretion. Related to Figure 1. (TIF) [file pone.0019414.s001.tif]

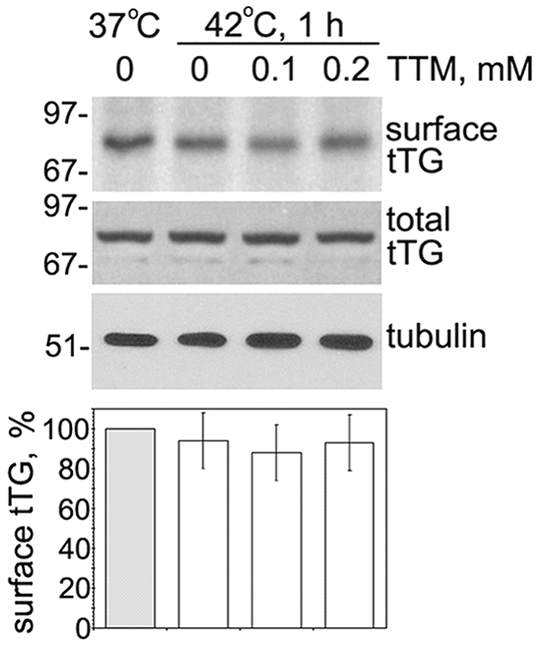

Supplement: Figure S2 — Heat shock and Cu2+ chelator do not affect tTG externalization. NIH3T3-tTG fibroblasts were treated for 18 h with 0-0.2 mM Cu2+ chelator ammonium tetrathiomolybdate (TTM) before inducton of tTG synthesis for 4 h. During the last hour of tTG synthesis, cells were left at 37°C or switched to 42°C. The surface tTG levels were defined after cell surface biotinylation and isolation of surface proteins (see Materials and Methods) by immunoblotting of cell surface protein fraction. The relative tTG levels on the surface of NIH3T3-tTG fibroblasts were compared with those in untreated cells at 37°C. Shown is representative of three independent experiments. Bars depict mean values ± SEM. The total tTG and tubulin levels were defined by direct immunoblotting. Note that heat shock and alteration of cellular Cu2+ levels, which affect the non-classical secretion of FGF1 [36], do not alter tTG externalization. Related to Figure 2. (TIF) [file pone.0019414.s002.tif]

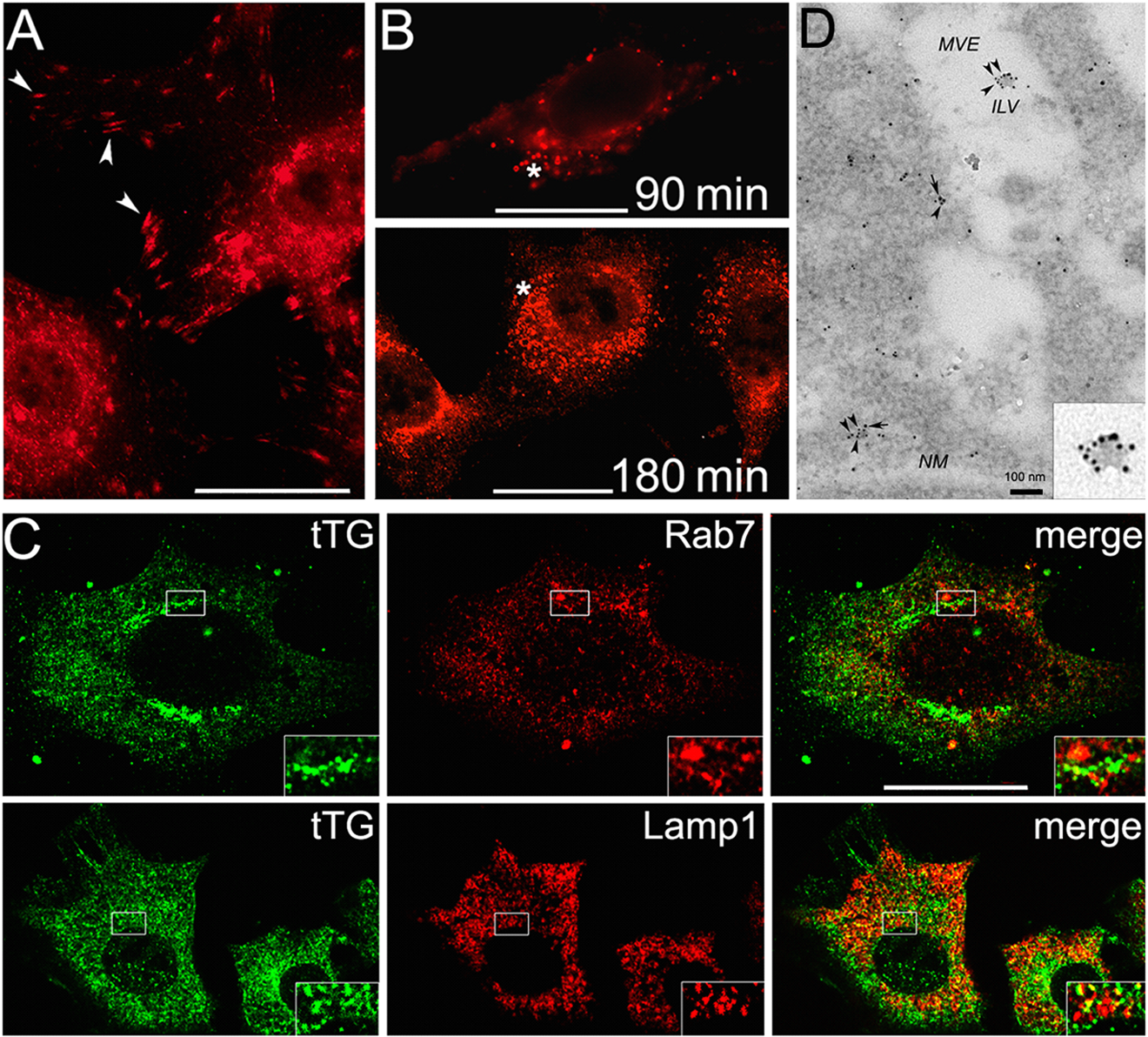

Supplement: Figure S3 — Intracellular localization of tTG in fibroblasts. (A,B) NIH3T3-tTG-His/Myc fibroblasts [39] were induced to synthesize His/myc-tagged tTG for 24 h (A) or indicated time (B). Cells were either fixed and permeabilized with formaldehyde and Triton X-100 (A), or extracted with digitonin before fixation (B), and then stained for tTG with antibody to 6xHis tag. Note tTG localization of in focal adhesions (arrowheads, (A)) and in perinuclear vesicles (asterisks, (B)). Immunofluorescence was analyzed by conventional microscopy. (C,D) NIH3T3-tTG fibroblasts were induced to synthesize tTG for 3 h. (C) Digitonin-extracted cells were double-stained for tTG and the late endosomal marker Rab7, or lysosomal marker Lamp1. Inserts show magnified perinuclear areas. Note a general lack of tTG co-localization with late endosomes and lysosomes. Immunofluorescence was analyzed by laser confocal microscopy. Bars - 10 μm. (D) Immunoelectron microscopic localization of tTG in NIH3T3-tTG fibroblasts. Double labeling of thin sections was performed for tTG (6 nm gold, arrowheads) and Rab11 (10 nm gold, arrows). NM - nuclear membrane; MVE - multivesicular endosome; ILV - intraluminal vesicle, also is shown as insert at higher magnification. Note the localization of tTG inside multivescular endosome on intraluminal vesicle. Related to Figure 3. (TIF) [file pone.0019414.s003.tif]

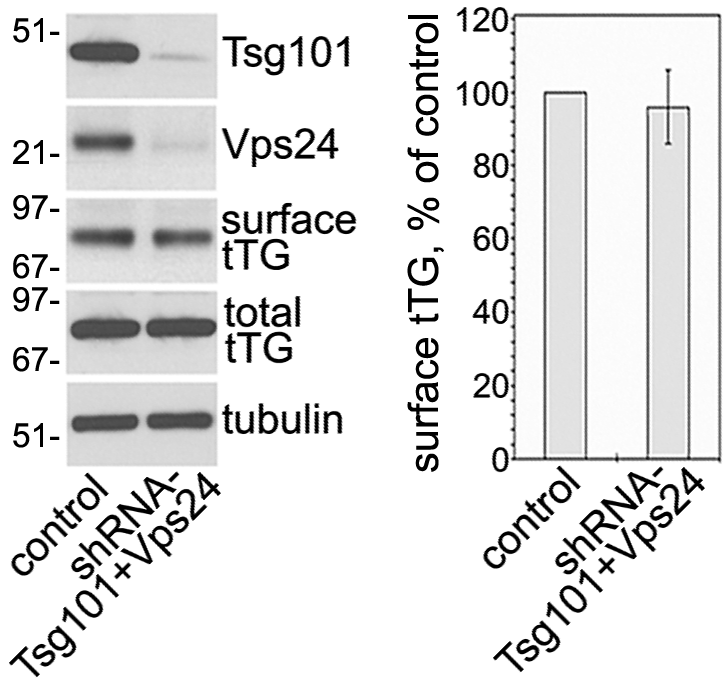

Supplement: Figure S4 — ESCRT function is not involved in tTG secretion. Depletion of Tsg101 and Vps24, the components of ESCRT-I and ESCRT-III complexes, respectively, was achieved by simultaneous transfection of shRNAs for these proteins into NIH3T3-tTG fibroblasts. tTG synthesis in these and control transfectants expressing scrambled shRNAs was induced for 4 h prior to cell surface biotinylation and isolation of surface proteins. Cell surface tTG levels and total levels of TSG101, Vps24, tTG, and tubulin were defined by immunoblotting. The relative surface level of tTG in the TSG101-, Vps24-depleted cells was compared to that in control transfectants expressing scrambled shRNAs. Shown is a representative of three independent experiments. Bars show means ± SEM. Related to Figure 3. (TIF) [file pone.0019414.s004.tif]

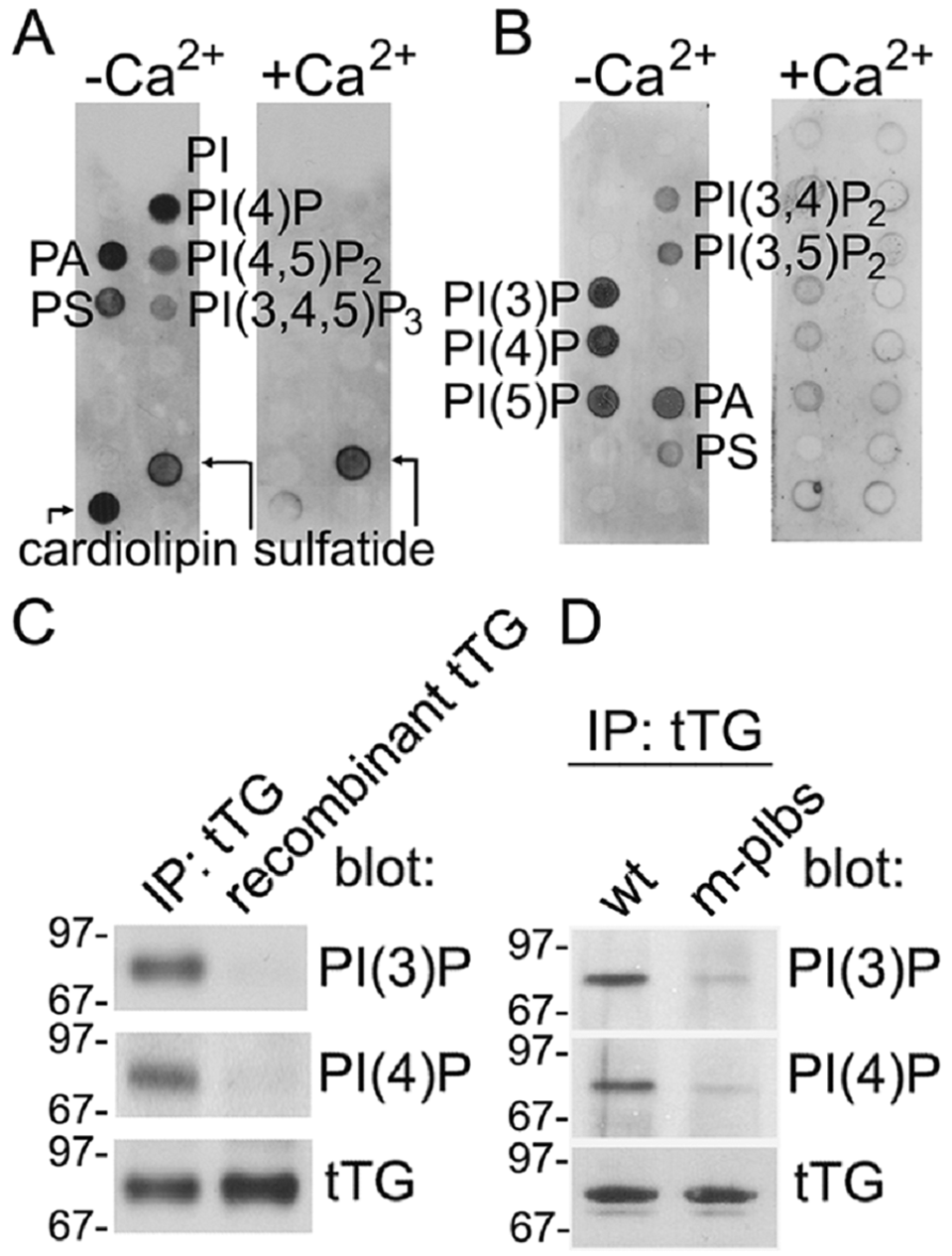

Supplement: Figure S5 — The interaction of tTG with phospholipids in vitro and in cells. (A,B) Interaction of tTG with phospholipids in vitro. Specificity and Ca2+ dependence of the interaction of tTG with membrane lipids (A) and phospholipids (B) in vitro was studied with membrane arrays (Echelon Biosciences). Bound tTG was detected by immunoblotting. (C,D) Interaction of tTG with phospholipids in cells. (C) tTG was immunoprecipitated from extracts of WI-38 fibroblasts. The resulting immune complexes and recombinant tTG purified from E. coli (Zedira) were analyzed by SDS-PAGE and immunoblotting with antibodies against PI(3)P and PI(4)P. Only the endogenous protein from fibroblasts, but not the recombinant tTG binds phosphoinositides. (D) Mutation of the presumed phospholipid-binding site interferes with the tTG-phosphoinositide association in fibroblasts. Wild type (wt) and K598A,K600A,R601A,K602A (m-plbs) mutant were expressed in NIH3T3 fibroblasts, then immunoprecipitated from cell extracts and tested for bound phospholipids by immunoblotting with antibodies against PI(3)P or PI(4)P. Shown in (A-D) are representative of three independent experiments. Related to Figure 6. (TIF) [file pone.0019414.s005.tif]
